# Hiatal Hernia in an Elderly Patient Complicated by Upper Gastroesophageal Bleeding, Pulmonary Embolism, and Respiratory Failure: A Case Report

**DOI:** 10.7759/cureus.76476

**Published:** 2024-12-27

**Authors:** Ali Al Ahmed, Fatema M Dubai, Hawra H Albasri, Tahera J Ahmed

**Affiliations:** 1 Internal Medicine, Salmaniya Medical Complex, Manama, BHR

**Keywords:** general internal medicine, hiatal hernia, pulmonary embolism, type ii respiratory failure, upper gastrointestinal bleed

## Abstract

Hiatal hernias occur when abdominal contents protrude into the posterior mediastinum through the esophageal hiatus of the diaphragm. They are classified into four types, with Type I (sliding) being the most prevalent. We report a case of a patient diagnosed with a large Type IV paraesophageal hernia. The patient presented with severe symptoms, including coffee ground vomiting and subsequent pulmonary embolism. Despite initial management, the patient's condition deteriorated, leading to respiratory failure. This case highlights the rare complications associated with paraesophageal hernias, particularly the risks of gastrointestinal bleeding and pulmonary complications. It underscores the importance of early recognition and intervention to prevent severe outcomes, such as respiratory failure. Hiatal hernias, though common, can lead to significant morbidity when complications arise. Clinicians should maintain a high index of suspicion for such complications in patients presenting with atypical symptoms.

## Introduction

A hiatal hernia occurs when abdominal contents protrude into the posterior mediastinum through the esophageal hiatus in the diaphragm. There are four types of hiatal hernias. Type I, or sliding hernia, involves the herniation of the cardiac orifice and the adjacent stomach. Type II, also known as pure paraesophageal or rolling hernia, is characterized by part of the stomach herniating anterior to the esophagus while the esophagogastric junction remains below the diaphragm. Type III is a combination of Type I and Type II. Finally, Type IV is a giant paraesophageal hernia that includes the herniation of the stomach along with other organs, such as the spleen, colon, or small intestine.

Hiatal hernias are relatively common, with Type I sliding hernias comprising approximately 95% of cases. Types II, III, and IV account for the remaining 5-15%, with giant paraesophageal hernias being particularly rare. The most frequent complication associated with hiatal hernias is gastroesophageal reflux [1]. However, giant paraesophageal hernias can lead to more serious complications, including ulceration, bleeding, strangulation, and respiratory issues, as research showed 14% of hernias are complicated by pulmonary manifestations. Here, we present a case of a large paraesophageal hernia that resulted in coffee ground vomiting, pulmonary embolism, and, ultimately, respiratory failure [2-3].

## Case presentation

An 83-year-old female with a history of congenital diaphragmatic hernia and osteoporosis presented to the emergency department in January 2024. She reported experiencing coffee ground vomiting twice and generalized abdominal tenderness. Upon arrival, she was found to be hypotensive, hypoxic, and tachycardic.

After stabilization, a chest X-ray (CXR) and chest computed tomography angiogram (CTA) were conducted to rule out surgical abdomen and mesenteric ischemia, leading to her admission under general medicine. Laboratory tests revealed an increased level of white blood cell count with the left shift. Blood and urine cultures returned negative, and she was initiated on antibiotics. Due to the huge distension of the stomach that was inflated and pushing the lungs, causing a transient restrictive disease, the patient was hypoxic. Thus, she was kept on oxygen.

**Figure 1:**
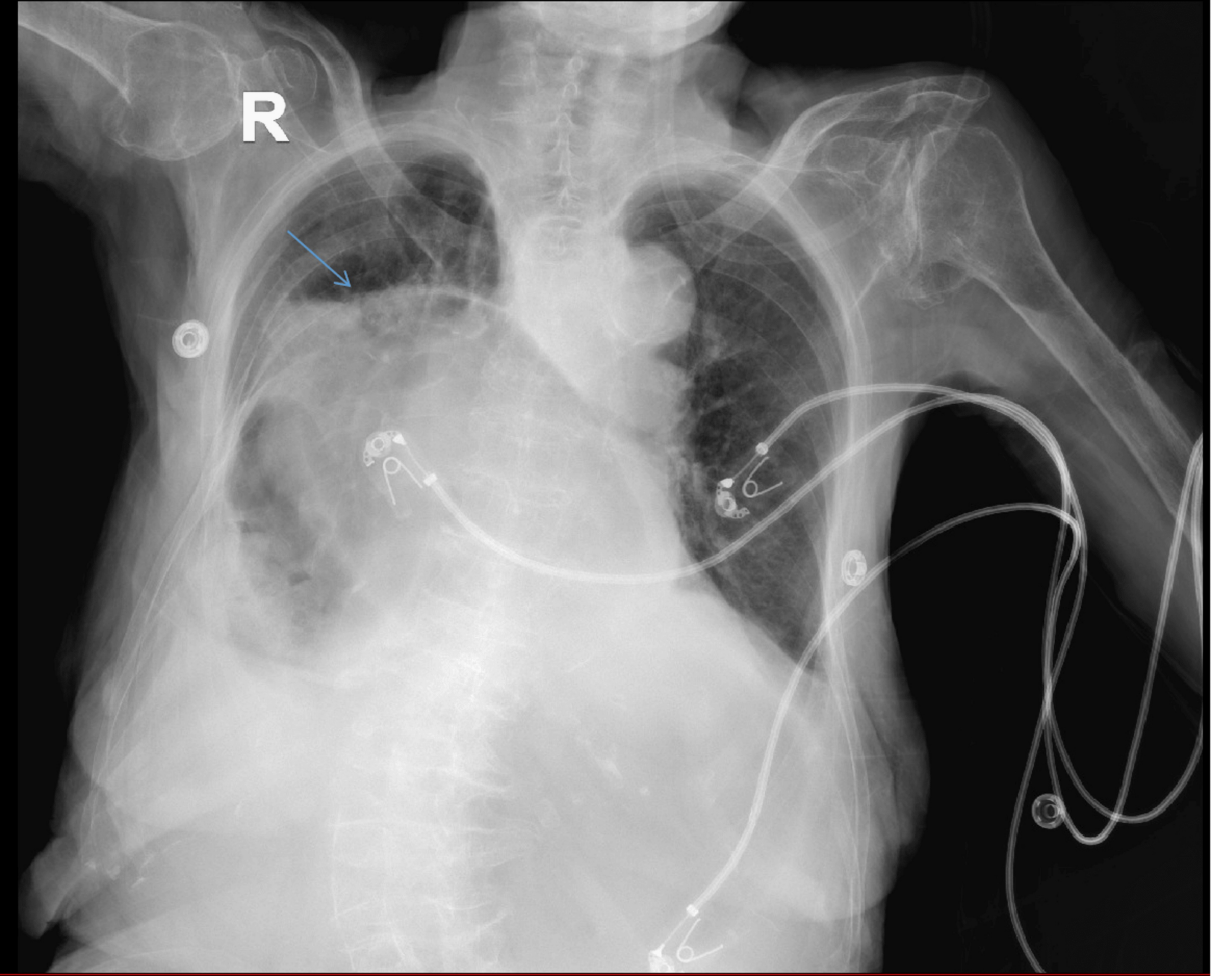
CXR on admission showing diaphragmatic hernia (arrow) containing a small loop of bowel, a dilated stomach, proximal duodenum, and ascending colon CXR: chest X-ray

**Figure 2:**
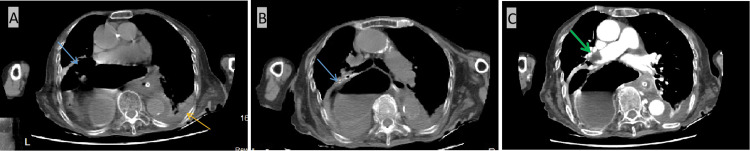
A-B: Chest CT on admission showing a large hiatal hernia involving the stomach and large bowel (blue arrow) with moderate pleural effusion (yellow arrow). C: CT pulmonary angiogram showing a filling defect in the right pulmonary artery suggestive of thrombus (green arrow) CT: computed tomography

The CXR indicated a diaphragmatic hernia containing a small loop of bowel, a dilated stomach, a proximal duodenum, and an ascending colon (Figure 1). The CTA (Figure 2) confirmed a large hiatal hernia involving the stomach and large bowel, along with a filling defect in the right main pulmonary artery extending to the segmental branches of the right lower lobe, indicative of pulmonary embolism. Moderate pleural effusion and compressive atelectasis of the right lower lobe were also observed due to the mass effect of the hernia.

A nasogastric tube (NGT) was placed, draining 2 lL of coffee ground vomitus. The pulmonology team advised against anticoagulation until bleeding ceased, while a Doppler ultrasound was planned. Concurrently, the gastroenterologist scheduled an esophagogastroduodenoscopy (OGD) and initiated an omeprazole infusion.

The bedside OGD revealed a large paraesophageal hernia with multiple ulcerations in the hernia part but no active bleeding. Ten days later, the patient experienced desaturation and decreased consciousness, responding only to painful stimuli. Her oxygen saturation was measured at 87% on 3 L of nasal cannula, with a mean arterial pressure of 65 while on dopamine (0.2 mg).

Since the patient had chronic constipation, two weeks after admission, she was found to be confused and progressed to respiratory failure, and her arterial blood gas (ABG) results are shown in Table 1. She was treated with laxatives and improved.

**Table 1:** ABG ABG: arterial blood gas

ABG	Value	Normal range
PH	7.35	7.35-7.45
PCO2	66	35-45 mmHg
PO2	77	75-11 mmHg
SPO2	99	94-100%
HCO3	32	22-26 mEq/L

**Figure 3:**
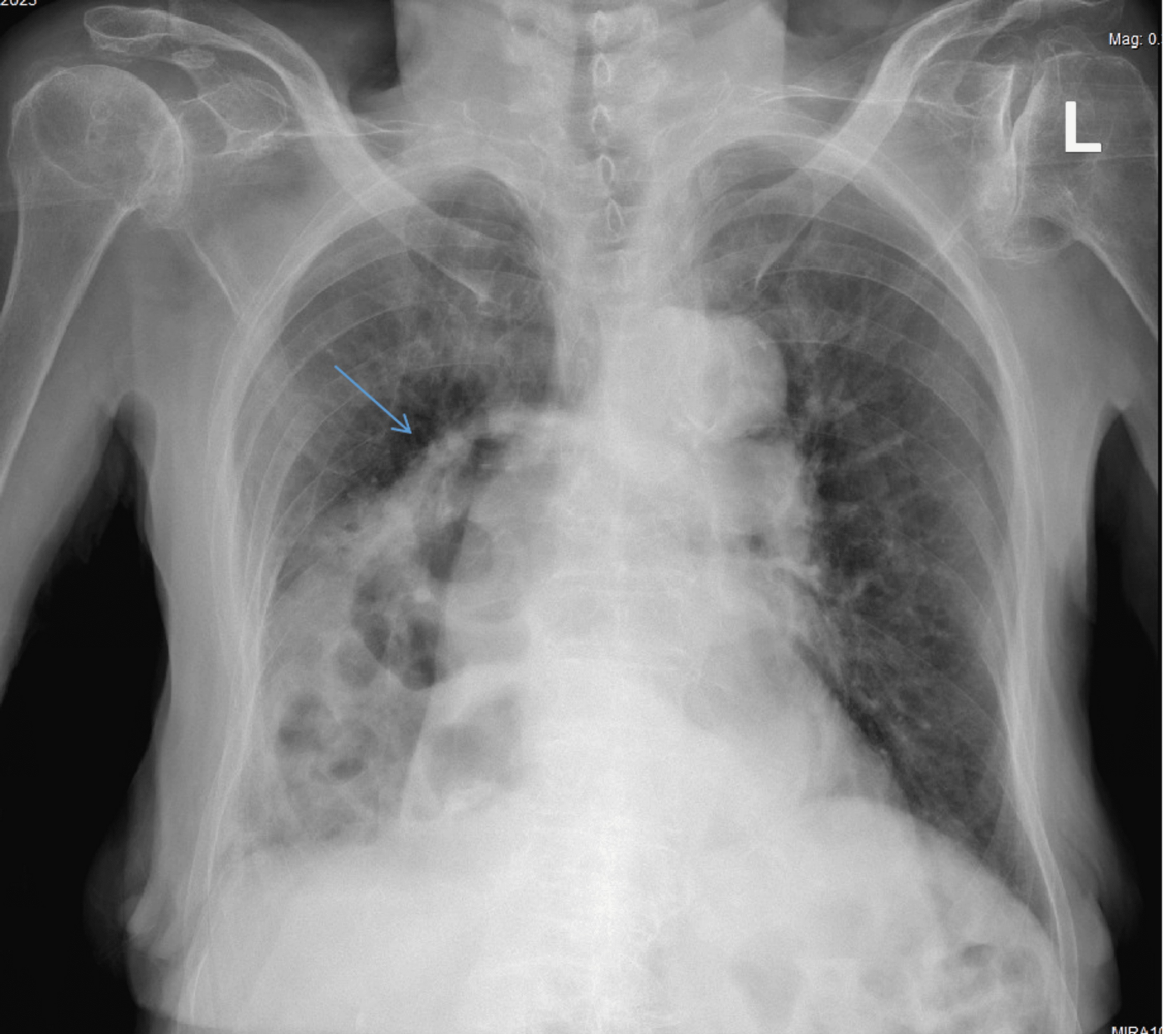
CXR before discharge showing the hiatal hernia after conservative management (arrow) CXR: chest X-ray

A subsequent chest CT scan revealed bilateral pleural effusion extending to the apex, collapse, and consolidation in the lower lobe with air bronchograms, an enlarged heart, diffuse aortic and valve calcification, and a dilated pulmonary trunk, suggesting pulmonary hypertension due to the main pulmonary embolism and gastric herniation into the right hemithorax. The patient was started on low-dose loop diuretics for pleural effusion. Eventually, the patient was stabilized, and CXR was repeated upon discharge (Figure 3).

She was discharged in good condition, without NGT, with an oxygen saturation of 98% on room air. Relatives were instructed on regimen laxatives that were prescribed, along with dietary follow-up, to avoid foods that could induce gas or constipation, thereby minimizing the risk of inflation and compression.

## Discussion

Severe complications associated with hiatal hernias, such as respiratory issues, pulmonary embolism, local thrombosis, and upper gastrointestinal bleeding, are relatively rare. Typically, hiatal hernias are clinically silent or manifest with gastroesophageal reflux symptoms, such as burning sensations. However, these rare complications can be life-threatening, necessitating immediate recognition and management.

In this case, the patient, with a virgin abdomen and no prior surgical history, exhibited symptoms of a congenital paraesophageal hiatal hernia, including coffee ground vomiting and hypoxia. The incidental finding of a pulmonary embolism contributed to her respiratory failure. The coffee ground vomiting stemmed from the organo-axial rotation of the stomach, leading to gastric ulceration due to acidity and localized ischemia, resulting in mucosal congestion and upper gastrointestinal bleeding. Additionally, the substantial hernia caused distension of the stomach and intestines, compressing the lungs and leading to restrictive lung disease [4,5].

Chronic pressure from the hernia on pulmonary vessels, combined with the patient's immobilization, contributed to blood stasis and ultimately resulted in pulmonary embolism, exacerbating hypoxia. Overall, giant paraesophageal hernias act as space-occupying lesions that exert pressure on adjacent structures. Early recognition and repair are crucial to prevent life-threatening complications.

Surgical repair of the hiatal hernia results in complete resolution of the respiratory complications; however, in this case, due to the age impact, treating the patient with NGT to reduce the inflation and compression on the lungs has excellent symptomatic results [6-8].

## Conclusions

This case highlights the challenges faced in managing a patient with a hernia, particularly in the context of age and comorbidities that rendered her unfit for laparoscopic repair, the preferred surgical intervention. Instead, a conservative treatment approach was implemented to alleviate her condition and prevent further complications. This included discharge with supplemental oxygen to address her increased respiratory needs resulting from hernial compression, reflecting the necessity of monitoring and adapting to her physiological requirements. Furthermore, a regimen of laxatives was prescribed alongside dietary guidance aimed at avoiding gas-producing or constipating foods. This strategy aimed to enhance the patient's comfort and sought to reduce the risk of hernia-related complications by minimizing the risk of inflation and compression, underscoring the importance of a multidisciplinary approach in managing complex cases where surgical options are limited.
